# Impact of lufenuron on hematology, serum biochemistry, antioxidant enzymes, and histopathology in broiler chickens

**DOI:** 10.1371/journal.pone.0319157

**Published:** 2025-03-26

**Authors:** Abu Baker Siddique, Riaz Hussain, Zeeshan Ahmad Bhutta, Muhammad Zishan Ahmad, Iahtasham Khan, Sana Alam, Rabbiah Manzoor Malik, Ayaz Mammadov, Farid Shokry Ataya

**Affiliations:** 1 Institute of Microbiology, Government College University, Faisalabad, Pakistan; 2 Department of Pathology, Faculty of Veterinary and Animal Sciences, The Islamia University of Bahawalpur, Bahawalpur, Pakistan; 3 Laboratory of Biochemistry and Immunology, College of Veterinary Medicine, Chungbuk National University, Cheongju, South Korea; 4 Faculty of Veterinary and Animal Sciences, PMAS Arid Agriculture University, Rawalpindi, Pakistan; 5 Department of Clinical Sciences, University of Veterinary and Animal Sciences, Sub-campus, Jhang, Pakistan; 6 Department of Zoology, The Islamia University of Bahawalpur, Bahawalpur, Pakistan; 7 Wah Medical College, Wah Cantt, Pakistan; 8 Department of Life Sciences, Western Caspian University, Baku, Azerbaijan; 9 Department of Biochemistry, College of Science, King Saud University, Riyadh, Saudi Arabia; Beni Suef University Faculty of Veterinary Medicine, EGYPT

## Abstract

The widespread utilization of synthetic chemicals, including weedicides, pesticides, insecticides, and fertilizers, has contaminated the environment. Therefore, it is crucial to recognize the ongoing threat of various stressors, including exposure to synthetic and natural chemicals on various target and non-target living organisms. Lufenuron is extensively utilized in controlling ectoparasites in companion animals and may offer a potential solution for addressing analogous challenges in commercial poultry farming. For this purpose, an experimental study was conducted to estimate the potential toxic effects of lufenuron in chickens. A total of 75 broiler chickens were housed under standard environmental conditions for the period of 39 days following a 7-day acclimatization. Birds were randomly divided into five groups (A-E), each consisting of 15 birds, and administered different doses of lufenuron in groups B-E @ 4, 8, 12, and 16 mg/kg body mass, respectively. Various hematological, biochemical, and histopathological biomarkers were assessed in blood and visceral organs. In the current experimental trial, the values of RBC count, PCV, hemoglobin, and MCHC significantly (<0.05) decreased, while WBCs and MCH significantly (<0.05) increased in treated birds. Results showed significantly increased values of renal function tests (urea and creatinine), liver function tests (ALP, AST, and ALT), and cardiac biomarkers (cholesterol and creatinine kinase) in broilers exposed to higher doses of lufenuron. Various histopathological ailments were examined in the liver, kidneys, and heart of the broiler in the dose and time-dependent manner. The current study showed that lufenuron induces oxidative stress, depletion of various antioxidant enzymes, and histoarchitectural alterations in multiple visceral organs.

## 1. Introduction

The poultry industry is an essential sector when it comes to food security for human beings all over the world, providing a tremendous source of protein. Poultry farming has become an increasingly vital part of agriculture in Pakistan, thus playing a vital role in both the country’s economy and food supply [1]. It is the leading country in poultry meat production among global leaders, and broiler chickens are the most reared kind of birds in the country because of their high growth rate and efficient feed conversion ratio. These are some of the facts that make the demand for poultry and its products in Pakistan since there is an increase in population, urbanization, and a shift towards meat consumption.

While advancements in genetics, nutrition, and management practices have propelled the poultry industry forward, it is essential to acknowledge the persistent threat posed by various stressors, including exposure to chemical agents [2,3]. Aside from the oxidative stress and lipid peroxidation that insecticides induce, they also alter the biochemical pathways in living organisms [4–7]. However, in terms of the developmental origins of health and disease, exposure to pesticides induces transgenerational effects. Monitoring of toxicological mechanisms of routinely and commonly used synthetic and environmental pollutants plays a vital role in limiting the adverse effects in non-target organisms [8–11].

Various synthetic chemicals, including fertilizers, pesticides, weedicides, and insecticides, are persistently utilized in agriculture, fruits, and vegetables to enhance crop production and control different pests [12–14]. Lufenuron, a member of the benzoylurea class of insect growth regulators, is primarily recognized for its role as a pesticide and insecticide [15]. It functions by inhibiting the synthesis of chitin, a critical component of the exoskeleton in insects, thereby disrupting their growth and development. This mode of action makes lufenuron particularly effective against a range of pests, including fleas and certain agricultural insects without affecting non-target species such as mammals and birds. Its application in agriculture has raised interest in understanding its potential impacts on non-target organisms, including poultry.

Hematological parameters stand as sentinel indicators of the physiological status of avian species, furnishing essential information on parameters such as red blood cell count, white blood cell count, and differential leukocyte counts [16]. Likewise, serum biochemistry profile, encompassing markers like total protein, albumin, globulin, and various enzyme are pivotal tools in evaluating organ functions and overall metabolic health [17]. In light of its potential influence on the oxidative stress response, investigating the impact of lufenuron on antioxidant enzyme assumes heightened significance [18]. Oxidative stress arises from an imbalance between the production of reactive oxygen species (ROS) and the organism’s capacity to neutralize them through antioxidant defenses [19]. Given the susceptibility of broiler chickens to oxidative stress, understanding how lufenuron may influence the antioxidant enzyme system holds the key to unraveling its broader physiological effects [20]. Histopathological examination of vital organs offers a microscopic perspective on tissue alterations, providing crucial information on the structural integrity and potential pathological changes occurring within these organs [21]. Understanding the histopathological effects of lufenuron on key organs is essential for assessing its overall impact on broiler health [22]. While lufenuron’s potential benefits in parasite control are well-documented, there remains a tenacious need to systematically examine its potential adverse effects, especially in broiler chicken production [23]. This study seeks to elucidate the toxic effect of lufenuron on hematology, serum biochemistry, histopathology, and antioxidant enzyme activities in broiler chickens. Through a comprehensive assessment of these parameters, we aim to provide a thorough understanding of the potential hazards associated with lufenuron exposure, shedding light on its potential risks to broiler health and welfare.

## 2. Materials and methods

### 2.1. Birds management and feed

A total of 75 broilers with similar body weight were reared at a local poultry farm in Bahawalpur, Pakistan, and were maintained under strict biosecurity and sanitation measures. All birds were housed in standard environmental conditions, with temperature at 26-28°C and humidity at 60-65%. Following a 5-day acclimatization period, the birds were randomly divided into five groups, each consisting of 15 chickens. The broilers received a diet containing 25% protein and had access to clean, fresh water daily.

### 2.2. Experimental design

Seventy-five male broilers were divided into five groups containing fifteen birds (n = 15/group). Group A served as the control, while groups B, C, D, and E were administered lufenuron orally at doses of 4, 8, 12, and 16 mg/kg body weight, respectively. The experimental period spanned 39 days, with sampling conducted on days 13, 26, and 39 of the study. All the experimental procedures were executed according to the guidelines and welfare regarding the use of laboratory animals of The Islamia University of Bahawalpur, Pakistan (No. 21-1051).

### 2.3. Blood sampling

Employing a sterile disposable syringe, blood samples for hematological and serum biochemical analyses were drawn from the wing vein of each broiler on the 13, 26, and 39^th^ day of the study [24]. Before weighing and having blood samples collected, the birds were deprived of feed and water for about two hours. Using a sterile syringe, approximately 3.0 mL blood was drawn from the wing vein and promptly transferred (0.5ml) into EDTA-coated vials for hematological analysis, while 2.5 mL of blood was collected without anticoagulant for serum biochemical analyses. The obtained serum was then preserved at -20°C for subsequent investigations. The birds were handled gently and with great care to minimize and alleviate stress, and to prevent any abrupt movements that could potentially lead to blood vessel damage or negative consequences [25]. After that, all the birds were sacrificed by cutting the Juglar vein.

### 2.4. Hematology and serum biochemistry

The hematological profile of the collected blood samples was assessed using established techniques. The collected samples underwent examination to ascertain values of red blood cells (RBCs), white blood cells (WBCs), hemoglobin (Hb), packed cell volume (PCV), mean corpuscular hemoglobin (MCH), and mean corpuscular hemoglobin concentration (MCHC) through the use of a specialized hematological analyzer [26]. Serum biochemical parameters including liver indicators such as aspartate aminotransferase (AST) (IU/L), alanine transaminase (ALT) (IU/L), and alkaline phosphatase (ALP) (IU/L), kidney biomarkers like creatinine (mg/dL) and urea (mg/dL) and cardiac biomarkers including creatinine Kinase MB (IU/L) and cholesterol (mg/dL), were assessed using a chemistry analyzer (Randox company Pvt.) [27].

### 2.5. Anti-oxidants parameters

Antioxidant enzymes, like Superoxide dismutase (SOD), Glutathione (GSH), peroxidase (POD), and Catalase (CAT) were quantified in various visceral tissues, including the heart, liver, and kidneys according to previous protocols [28–30].

### 2.6. Histopathology

The heart, kidneys, and liver tissues were promptly removed from both the treated and control birds for histopathological alterations. These tissues were then preserved in a 15% formaldehyde solution. Following fixation, all the tissues underwent a sequence of procedures, including washing, dehydration using increasing ethanol concentrations, clearing with xylol, and embedding in molten paraffin wax [31]. Multiple sections of each specimen were obtained by rotary microtome, and a minimum of two slides, each containing three to four sections, were processed. Subsequently, these slides were stained with hematoxylin and eosin and examined under a light microscope [31,32].

### 2.7. Statistical analysis

To assess the differences in hematology, biochemical parameters, and antioxidant enzymes among various groups, the mean values derived from replicates underwent statistical analysis using Analysis of Variance (ANOVA) techniques. Tukey’s test was employed to compare the means of distinct groups, with a significance level set at P < .05. This threshold was considered indicative of statistical significance.

## 3. Results

### 3.1. Hematology

**Fig 1:**
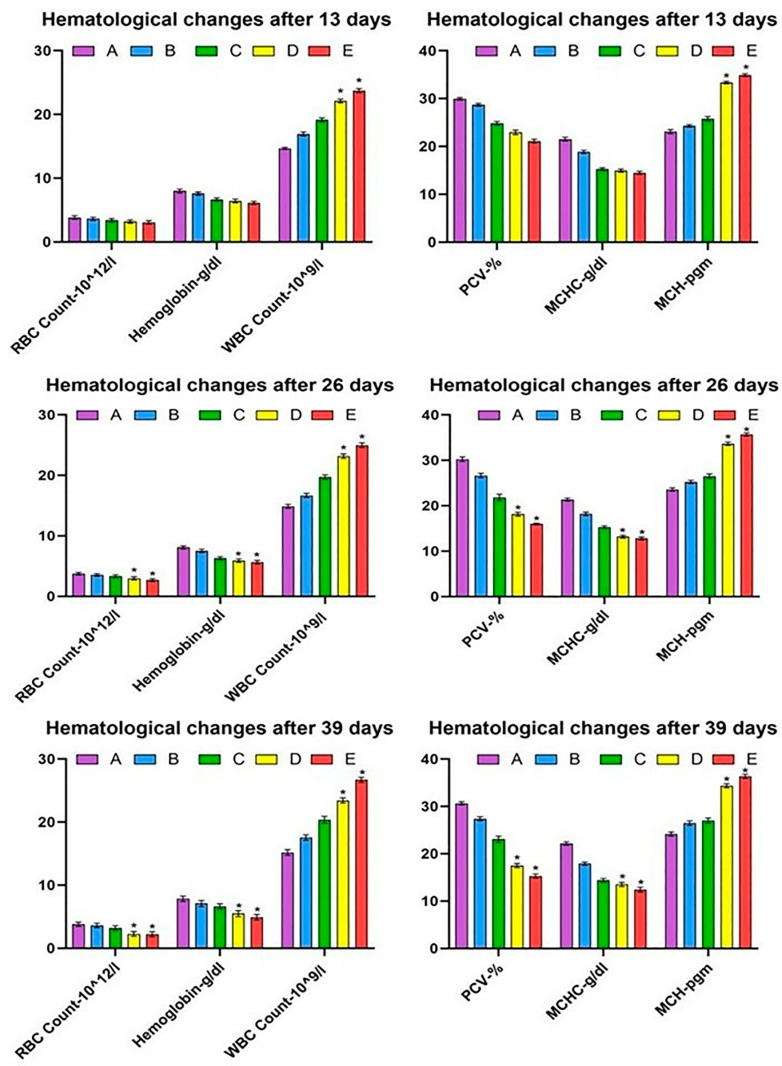
Comparison of various hematological Profile of broilers administered different doses of lufenuron. Bars indicate values (mean ± SE), while *  indicates significant difference at p < 0.05. Data obtained from each group (mean±SE) was subjected to Turkey’s post hoc test.

The values of RBC count in broilers administered with different doses of lufenuron in different groups are indicated in Fig 1. The results showed that RBC counts in the treated broiler was significantly (<0.05) different from the broiler kept in the control group. The RBC count, PCV, hemoglobin, and MCHC significantly (<0.05) decreased at higher doses in groups D-E at 26 and 39 days of the experiment. The values of WBCs and MCH significantly (<0.05) increased in groups D-E at days 26 and 39 of the experiment.

### 3.2. Serum biochemistry

**Fig 2:**
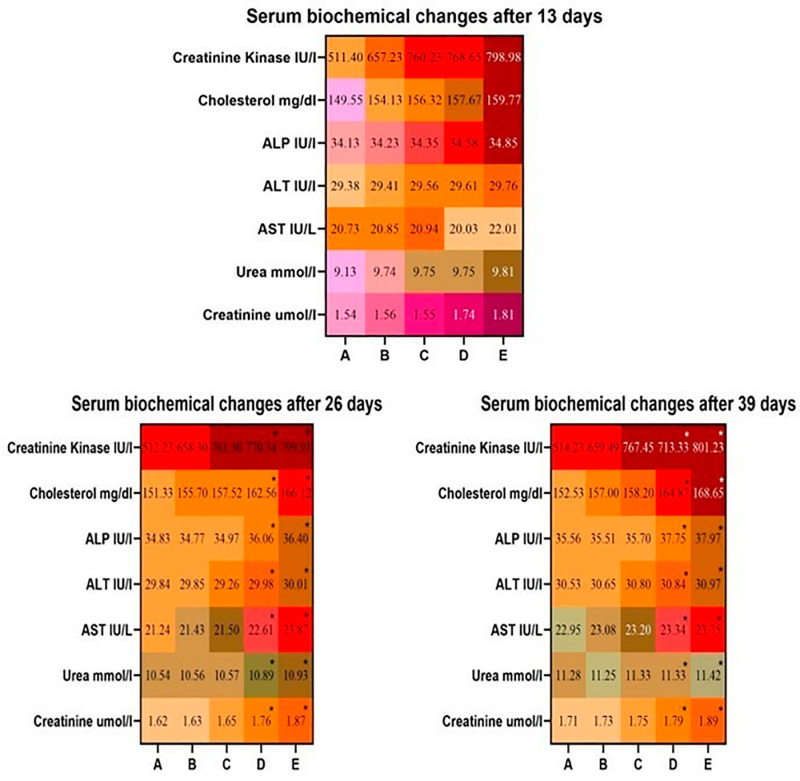
Comparison of various biochemical profile of broilers administered different doses of lufenuron. * p < 0.05, Data obtained from each group (mean±SE) was subjected to Turkey’s post hoc test.

The results showed significant alterations in the serum biochemistry of broilers exposed to higher concentrations of lufenuron, especially in groups D and E, as compared to broilers of the control group (Fig 2). A significant increase in values of liver biomarkers including ALT, AST, and ALP, was observed in broilers of groups D and E on days 26 and 39 of the study.. A substantial augmentation in the concentration of kidney biomarkers including urea and creatinine was evaluated in groups D and E on days 13, 26, and 39 of the study. However, values of heart biomarkers, including cholesterol and creatinine kinase showed a remarkable increase in broilers of groups D and E at days 26 and 39 of the study.

### 3.3. Status of antioxidants and oxidative stress parameters in the heart, liver, and kidneys

**Fig 3:**
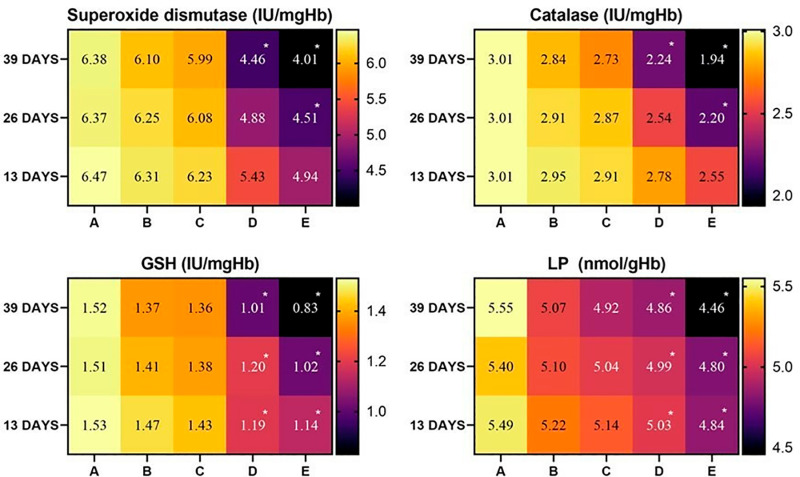
Status of various antioxidants in heart tissue of broiler exposed to various concentrations of lufenuron. * p < 0.05, Data obtained from each group (mean±SE) was subjected to Turkey’s post hoc test.

The concentrations of oxidative stress parameter POD and non-enzymatic antioxidant GSH showed a similar patterns of alteration in heart tissues of the broiler during the whole experimental procedure. It was observed that the values of POD and GSH decreased in heart tissues of groups D and E on days 26 and 39 of the study. Catalase and SOD values decreased significantly in group D at day 39, while decreased significantly in group E at day 26 and 39 of the study (Fig 3).

**Fig 4:**
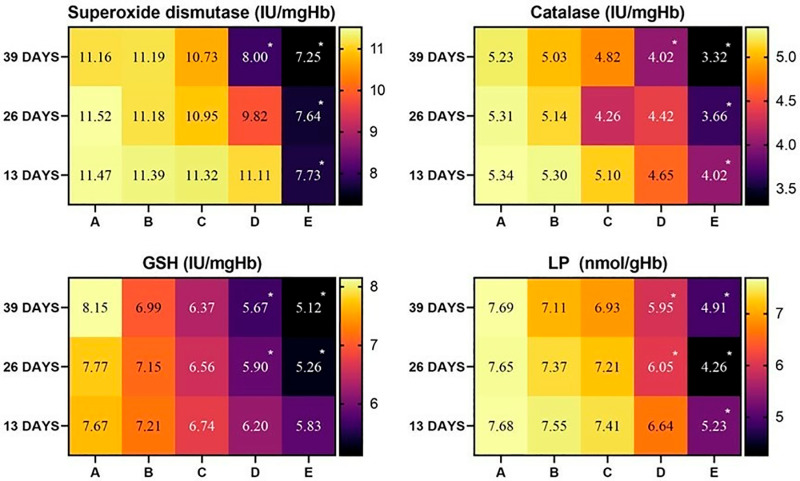
Status of various antioxidants in liver tissue of broiler exposed to various concentrations of lufenuron. * p < 0.05, Data obtained from each group (mean±SE) was subjected to Turkey’s post hoc test.

It was observed that the values of GSH decreased in liver tissues of group D and E at day 26 and 39 of the study. While POD values decreased significantly in group D at days 26 and 39, while the contents of POD decreased significantly in group E at days 13, 26, and 39 of the study. Catalase and SOD showed the same pattern of alteration. During the study, it was observed that CAT and SOD values decreased significantly in group D at days 26 and 39 while decreasing significantly in group E at days 13, 26, and 39 of the study (Fig 4).

**Fig 5:**
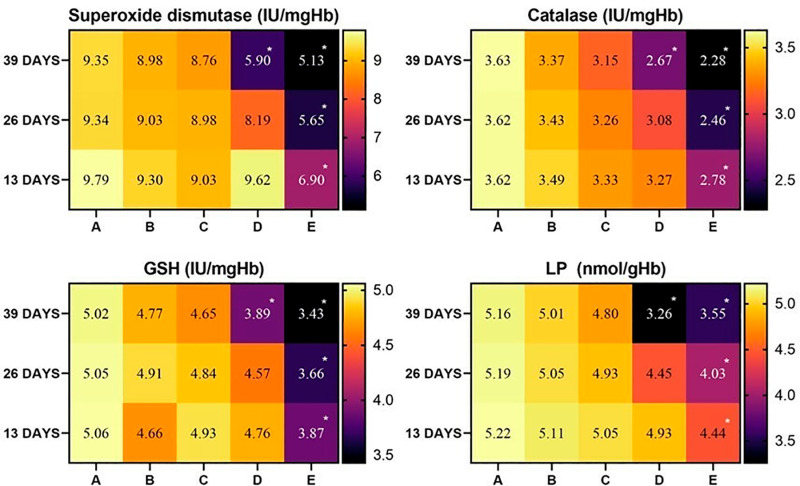
Status of various antioxidants in kidney tissues of broiler exposed to various concentrations of lufenuron. * p < 0.05, Data obtained from each group (mean±SE) was subjected to Turkey’s post hoc test.

In kidney tissues, the same pattern of alterations in the values of oxidative stress parameter (POD) and non-enzymatic antioxidant (reduced GSH) and enzymatic antioxidants (CAT and SOD) were observed during the study. It was observed that the concentrations of POD, GSH, CAT, and SOD decreased in the kidney tissues of broilers of group D at day 39 and group E at days 13, 26, and 39 of the study (Fig 5).

### 3.4. Histopathology

On days 26 and 39 of the experiment, distinct histological abnormalities were evident in the kidneys of broilers reared in groups E, including extensive necrosis of tubular cells, necrosis of tubular cells, widening to urinary space, edema, and degeneration and necrosis of renal tubules. Similar moderate histopathological observations in the kidneys of broilers from group D at day 26 while increased severity of histopathological alterations (extensive necrosis of tubular cells, tubular necrosis, edema, and degeneration of renal tubules) was observed at day 39 of the study. revealed. Various liver sections of different birds from group E at days 26 and 39 indicated various pathological abnormalities, including congestion, vacuolar degeneration, disorganization of hepatic cords, increased sinusoidal space, and necrotic hepatocytes. Conversely, similar moderate pathological lesions in the liver sections of broilers from group D at day 26 while severe at day 39 were observed. The histopathological examination of the heart in broilers from group D-E at day 39 showed severe pathological changes, including degeneration and disorganization of cardiac muscles, edema, necrosis of cardiac myocytes, breakdown of muscle fibers inflammatory material, exudate, and fatty infiltrations were observed in various sections of the hearts of broilers. On day 26 moderate histopathological lesions in group D at day 26 while severe at day 26 in broilers of group E were examined in the current study. Mild to moderate histopathological lesions in the liver, kidneys, and heart of broilers exposed to lufenuron of group C were examined on days 26 and 39 of the trial.

## 4. Discussion

Ensuring food security is a key sustainable development goal in many developing countries [33]. Lufenuron, a benzoylphenylurea, and a vital pesticide in the insect development inhibitor (IDI) family exhibits lasting environmental persistence with an average field half-life of 5-13 days [34]. Residues of lufenuron in soil, fruits, and various plant components pose a significant threat to ecological species in agricultural areas. Monitoring and evaluating these agrochemicals are crucial to reduce harmful effects on public health, as studies suggest that pesticides can induce gonadotoxic effects [2,35], genetic abnormalities, cancer, infertility, and prenatal deformities in non-target species [36–38].

In the current experimental trial, the values of RBC count, PCV, hemoglobin, and MCHC significantly (<0.05) decreased at a higher dose in group D and group E of broiler, while the values of WBCs, MCH significantly (<0.05) increased in group D and E of the current study. Similar results were observed in Japanese quail [39] and White Leghorn cockerels [3]. The lower hematological profile in bailors administered higher [3] doses of lufenuron could be due to toxic effects on red blood cells, hemolysis of erythrocytes in microvasculature, and physiological disturbances on blood forming tissues in bone marrow [2]. The increased values of total white blood cells might be due to the triggering of inflammation in multiple exposed tissues of broilers. Moreover, it is also recorded that various environmental pollutants/toxicants can trigger the complex process of inflammation, leading to the induction of oxidative stress in various systems of exposed living organisms [40]. The lower concentrations of hemoglobin and erythrocytes in treated birds may be due to the toxic effects of lufenuron on bone marrow and abnormal physiological disturbances in the kidneys and liver. The values of the biomarkers of kidneys (urea and creatinine), liver (ALP, AST, and ALT), and heart (cholesterol and creatinine kinase) showed significant augmentation in the serum of broiler exposed to higher doses of lufenuron. In previous studies, similar results were observed in Japanese quail [41], male albino rats [42] and adult cockerels [3,11]. The broilers exposed to various concentrations of lufenuron showed a significant decline in the values of various antioxidants (reduced GSH, CAT, SOD). Our results are best supported by literature, which reported a similar significant reduction of antioxidant enzymes in quails exposed to toxins [43,44]. The decrease in concentrations of antioxidant enzymes in our study might be related to the induction of oxidative stress by lufenuron in broilers. It is well established that SOD and CAT play essential and vital role in detoxifying hydrogen peroxide and superoxide radicals, while POD hunt lipid peroxides [45]. The adverse hepatic alterations in birds exposed to lufenuron could be due to being the primary site of normal metabolism and an important visceral organ involved in various detoxification mechanisms [46–48]. Previously, different studies have indicated that overproduction of free radicals due to exposure to toxicants induce oxidative stress associated with depletion of antioxidant enzymes resulting in poor performance of immune system of exposed organisms [49–51].

The depletion of these antioxidant enzymes impairs the neutralization capacity and thus enhances the induction of oxidative damage. Furthermore, the depletion of glutathione, glutathione-S-transferase, CAT, and SOD in the liver and kidneys of rodents due to oxidative stress has been recorded [52–54]. Moreover, overproduction and unregulated free radicals suppress CAT, SOD, and POD leading to biomolecular oxidation and cell injury [55]. Distinct histological abnormalities were evident in broilers’ kidneys, liver and heart. Kidneys exhibited necrosis of tubular cells, tubular necrosis, edema, and degeneration of renal tubules of broiler exposed to higher concentrations of lufenuron. Previously, it was observed in quails exposed to toxins [56]. Lufenuron induced various histopathological abnormalities, congestion, vacuolar degeneration, disorganization of hepatocytes, and necrotic hepatocytes in the liver of broilers. The microscopic lesions in the kidneys of broilers administered lufenuron at higher concentrations could be associated with the triggering of inflammation causing the generation of free radicals [2,6,8]. Previously, scanty information can be found in the kidneys and liver of broilers administered various doses of lufenuron. The microscopic lesions could be due to exposure to lufenuron, resulting in the activation of various prototypical AhR-regulated genes (Cyp1b1, Nqo1, Cyp1a1, and mRNA) responsible for the induction of microscopic ailments due to nuclear localization [57]. The histopathological lesions in multiple visceral organs could also be related to stimulation and activation of different pathways (TLR4/NF-κB/NLRP3 and TNFα/TNFR1), causing the formation of the complex (RIPK1/RIPK3) and activation of NLRP3 and Caspase-1 inflammasomes [58]. The histo-architectural changes in the kidneys of bailors administered higher levels of lufenuron could also be associated with increased release (interleukin-8) and alterations (interleukin-8 promoter) due to acetylation of histone and methylation of nuclear material. Furthermore, the necrotic changes in various visceral organs in our study, including the liver, kidneys, and heart, could be due to the induction of oxidative stress by lufenuron [8,10]. In previous studies, similar results were observed in *Collumba livia* [59] and Gallus birds [13](Khan, 2022). Degeneration, myofibrillosis, inflammatory exudate, and edema were observed in the heart tissue of broilers exposed to higher doses of lufenuron. Previous studies did not provide any published data on the histological changes in the hearts of broilers induced by lufenuron.

In conclusion, lufenuron has the potential to induce oxidative stress, depletion of various antioxidant enzymes, and histoarchitectural alterations in multiple visceral organs. Given these potential risks, poultry farmers should seek ways of carrying out pest management that will not interfere with the health of the animals. There are natural and organic methods being tried, like essential oils, probiotics, and organic acids, which have been found to enhance gut health and performance in broiler chickens. As an illustration, organic acidifiers could be anti-bacterial in nature and serve to enhance gut microbiota, thereby decreasing dependence on chemical pesticides like Lufenuron. Besides this, the inclusion of nutraceuticals in the form of antioxidants and herbal supplementation could enhance the immune system through a reduction in oxidative stress and also provide a new paradigm for poultry health management. Further chronic exposure analyses and searches for alternative active ingredients with the capability of providing good pest control without health and welfare implications for broilers should be carried out. By paying proper attention to animal health and sustainability in poultry production methods of pest control, increased productivity will be achieved in a manner that doesn’t compromise the safety and welfare of broiler chicken birds and consumers.

## References

[1] Ahmad S, Humak F, Ahmad M, Altaf H, Qamar W, Hussain A. Phytochemicals as alternative anthelmintics against poultry parasites: a review. 2023;12:34–45. 10.47278/journal.abr/2023.015

[2] Hussain R, Ali F, Javed MT, Jabeen G, Ghaffar A, Khan I, et al. Clinico-hematological, serum biochemical, genotoxic and histopathological effects of trichlorfon in adult cockerels. Toxin Reviews. 2019;40(4):1206–14. doi: 10.1080/15569543.2019.1673422

[3] Hussain R, Ghaffar A, Ali HM, Abbas RZ, Khan JA, Khan IA, et al. Analysis of different toxic impacts of Fipronil on growth, hemato-biochemistry, protoplasm and reproduction in adult cockerels. Toxin Reviews. 2017;37(4):294–303. doi: 10.1080/15569543.2017.1366921

[4] Ndonwi EN, Atogho-Tiedeu B, Lontchi-Yimagou E, Shinkafi TS, Nanfa D, Balti EV, et al. Gestational Exposure to Pesticides Induces Oxidative Stress and Lipid Peroxidation in Offspring that Persist at Adult Age in an Animal Model. Toxicol Res. 2019;35(3):241–8. doi: 10.5487/TR.2019.35.3.241 31341553 PMC6629439

[5] Al-Saeed FA, Naz S, Saeed MH, Hussain R, Iqbal S, Mustafa Chatha AM. Oxidative stress, antioxidant enzymes, genotoxicity and histopathological profile in Oreochromis niloticus exposed to lufenuron. Pakistan Veterinary Journal. 2023;43(1):.

[6] Kazmi SAH, Iqbal R, Al-Doaiss AA, Ali M, Hussain R, Latif F, et al. Azoxystrobin-induced Oxidative Stress in Gills, Hematological Biomarkers and Histopathological Ailments in Fresh Water Fish. Pakistan Veterinary Journal. 2023;43(2).

[7] Abbassy MA, Khalifa MA, Nassar AMK, El-Deen EEN, Salim YM. Analysis of organochlorine pesticides residues in fish from Edko Lake (North of Egypt) using eco-friendly method and their health implications for humans. Toxicol Res. 2021;37(4):495–503. doi: 10.1007/s43188-020-00085-8 34631506 PMC8476673

[8] Wang J-Q, Hussain R, Ghaffar A, Afzal G, Saad AQ, Ahmad N, et al. Clinicohematological, Mutagenic, and Oxidative Stress Induced by Pendimethalin in Freshwater Fish Bighead Carp (Hypophthalmichthys nobilis). Oxid Med Cell Longev. 2022;2022:2093822. doi: 10.1155/2022/2093822 35528506 PMC9072014

[9] Ahrar Khan AK, Riaz Hussain RH, Javed MT, Fazal Mahmood FM. Molecular analysis of virulent genes (coa and spa) of Staphylococcus aureus involved in natural cases of bovine mastitis. 2013.

[10] Ghaffar A, Hussain R, Ahmad N, Ghafoor R, Akram MW, Khan I, et al. Evaluation of hemato-biochemical, antioxidant enzymes as biochemical biomarkers and genotoxic potential of glyphosate in freshwater fish (Labeo rohita). Chemistry and Ecology. 2021;37(7):646–67. doi: 10.1080/02757540.2021.1937141

[11] Hussain R, Ghaffar A, Abbas G, Jabeen G, Khan I, Abbas RZ, et al. Thiamethoxam at sublethal concentrations induces histopathological, serum biochemical alterations and DNA damage in fish (Labeo rohita). Toxin Reviews. 2020;41(1):154–64. doi: 10.1080/15569543.2020.1855655

[12] Shafqat S, Ismail Y, Umar M, Shafqat D, Obaid M, Zan X-Q. Oxidative stress and toxicological impacts of Ethoxysulfuron exposure on bone marrow, and intestinal morphometry in male Japanese Quail. Continental Veterinary Journal. 2024;3(2):78–85.

[13] Khan A, Asrar R, Shrafat H, Qamar M, Ahmad S, Kauser M. Oxidative stress and toxicological impacts of Monomehypo exposure on bone marrow and erythrocytes in male Japanese quail. Continental Veterinary Journal. 2023;3(1):84–90.

[14] Kanwal N, Aziz S, Abdullah S, Ali M, Ahmad N. Continental veterinary journal. Continental Veterinary Journal. 2024;4(1):40–5.

[15] Imran M, Hanif K, Ahmad M, Nasir M, Ayyaz U, Sheikh UAA. Comparative Toxicity of Insecticides against Two Important Insect Pests of Cauliflower Crop. Asian Journal of Agriculture and Biology. 2017;5(2).

[16] García G, Paterlini C, Hernandez M, Behotas T, Favero M, Seco Pon J. Hematology and plasma chemistry values in beached Magellanic Penguin (Spheniscus magellanicus) in northern Argentina during the non-breeding season. Journal of Zoo and Wildlife Medicine. 2020;50:927–36. 31926525 10.1638/2019-0012

[17] Novotný J, Horáková L, Řiháček M, Zálešáková D, Šťastník O, Mrkvicová E, et al. Effect of Different Feed Particle Size on Gastrointestinal Tract Morphology, Ileal Digesta Viscosity, and Blood Biochemical Parameters as Markers of Health Status in Broiler Chickens. Animals (Basel). 2023;13(15):2532. doi: 10.3390/ani13152532 37570340 PMC10417443

[18] Basal W, Omar A, Abdel Nasser Mahmoud A. Exposure to Lufenuron during the third gestational period induces genotoxicity and oxidative stress effects in pregnant albino rats and their fetuses. Egypt Acad J Biol Sci. 2021;13:121–34.

[19] Adwas A, Elsayed A, Azab A, Quwaydir F. Oxidative stress and antioxidant mechanisms in human body. J Biotechnol. 2019;6:43–7.

[20] Chen Y, Han S, Wang Y, Li D, Zhao X, Zhu Q. Oxidative stress and apoptotic changes in broiler chicken splenocytes exposed to T-2 toxin. Biomed Research International. 2019;2019:5493870. doi: 10.1155/2019/5493870 31886226 PMC6925674

[21] Rebez EB, Sejian V, Silpa MV, Dunshea FR. Heat Stress and Histopathological Changes of Vital Organs: A Novel Approach to Assess Climate Resilience in Farm Animals. Sustainability. 2023;15(2):1242. doi: 10.3390/su15021242

[22] Junquera P, Hosking B, Gameiro M, Macdonald A. Benzoylphenyl ureas as veterinary antiparasitics. An overview and outlook with emphasis on efficacy, usage and resistance. Parasite. 2019;26:26. doi: 10.1051/parasite/2019026 31041897 PMC6492539

[23] Buchmann K. Control of parasitic diseases in aquaculture. Parasitology. 2022;149(14):1985–97. doi: 10.1017/S0031182022001093 35950444 PMC10090776

[24] Benjamin MM. Outline of veterinary clinical pathology. 3d ed. Ames: Iowa State University Press; 1978.

[25] Arora K. Differences in hemoglobin and packed cell volume in blood collected from different sites in Japanese quail (Coturnix japonica). Int J Poult Sci. 2010;9.

[26] Chanu KR, Mangang YA, Debbarma S, Pandey PK. Effect of glyphosate-based herbicide roundup on hemato-biochemistry of Labeo rohita (Hamilton, 1822) and susceptibility to Aeromonas hydrophila infection. Environ Sci Pollut Res Int. 2023;30(51):110298–311. doi: 10.1007/s11356-023-29967-8 37783989

[27] Mahmood Y, Ghaffar A, Hussain R, editors. New Insights into Hemato-Biochemical and Histopathological Effects of Acetochlor in Bighead Carp (Aristichthys nobilis). 2021.

[28] Beutler E, Duron O, Kelly BM. Improved method for the determination of blood glutathione. J Lab Clin Med. 1963;61:882–8. 13967893

[29] Aebi H. [13] Catalase in vitro. Methods in Enzymology. 105: Academic Press; 1984;105. p. 121–6. 10.1016/s0076-6879(84)05016-3 6727660

[30] Raza GA, Ghaffar A, Hussain R, Jamal A, Ahmad Z, Mohamed BB, et al. Nuclear and Morphological Alterations in Erythrocytes, Antioxidant Enzymes, and Genetic Disparities Induced by Brackish Water in Mrigal Carp (Cirrhinus mrigala). Oxid Med Cell Longev. 2022;2022:4972622. doi: 10.1155/2022/4972622 36267815 PMC9578798

[31] Mohammadzadeh P, Rasuli A, Shadan N, Najafi F, Bashiri A, Mohammadi S. Histopathological findings associated with capture myopathy in Persian onager (Equus hemionus onager). International Journal of Veterinary Science. 2023;12(3):401–6.

[32] Suvarna KS, Layton C, Bancroft JD. Bancroft’s theory and practice of histological techniques E-Book: Elsevier health sciences; 2018.

[33] Subedi D, Farhan M, Niraula A, Shrestha P, Chandran D, Acharya K. Avian influenza in low and middle-income countries (LMICs): outbreaks, vaccination challenges and economic impact. Pakistan Veterinary Journal. 2024;44(1).

[34] Hassan E, Ahmed N, Arief M. Dissipation and Residues of Lufenuron in Grape Fruits. American Journal of Environmental Protection. 2013;1(2):17–9. doi: 10.12691/env-1-2-1

[35] Shahid M, Shaukat F, Shahid A, Sohail A, Nadeem M. Biopesticides: a potential solution for the management of insect pests. Agrobiological Records. 2023;13:7–15.

[36] Gerken J, Vincent GT, Zapata D, Barron IG, Zapata I. Comprehensive assessment of pesticide use patterns and increased cancer risk. Front Cancer Control Soc. 2024;2. doi: 10.3389/fcacs.2024.1368086

[37] Maharjan A, Gautam R, Acharya M, Jo J, Lee D, K C PB, et al. Association of immunotoxicological indices with lung cancer biomarkers in poultry, grape, and rose farming workers. Toxicol Res. 2023;39(4):739–47. doi: 10.1007/s43188-023-00199-9 37779584 PMC10541357

[38] Ahmad L, Gul S, Kashif M, Hussain R, Rehman A, Naimul S. The effect of different repeated doses of cypermethrin on the behavioral and histological alterations in the brain of rabbits (Oryctolagus cuniculi). International Journal of Veterinary Science. 2021;10:347–54.

[39] Hussain R, Mahmood F, Khan A. Genotoxic and pathological effects of malathion in male Japanese quail (Coturnix japonica). Pak J Agri Sci. 2015;52(4):1149–56.

[40] Ji H. lncRNA NONRATT021477 interference aggravates H2O2-induced oxidative stress in BRL cells. Kafkas University Faculty of Veterinary Medicine Journal. 2024;30(6):779–85.

[41] Ghaffar A, Hussain R, Abbas G, Ali M, Ahmad H, Nawaz J. Arsenic and copper sulfate in combination causes testicular and serum biochemical changes in white leghorn cockerels. Pakistan Veterinary Journal. 2017;37(4).

[42] Ijaz M, Kalsoom A, Hamza A, Ehsan N. Sciadopitys attenuates paraquat induced renal toxicity by modulating Nrf-2/Keap-1 pathway in male albino rats. Asian J Agric Biol. 2023;10:2023110.

[43] Akram R, Ghaffar A, Hussain R, Khan I, de Assis Santana V, Mehmood K. Hematological, serum biochemistry, histopathological and mutagenic impacts of triclosan on fish (bighead carp). Agrobiological records. 2022;7:18–28.

[44] Rani A, Afzal G, Mahmood Y, Alam S, Iqbal Z, Akbar M. Ethoxysulfuron causes nuclear abnormalities in erythrocytes, DNA damage in some visceral organs, and oxidative stress in male Japanese quail. Asian Journal of Agriculture and Biology. 2023(3).

[45] Kumar N, Thorat ST, Reddy KS. Multi biomarker approach to assess manganese and manganese nanoparticles toxicity in Pangasianodon hypophthalmus. Sci Rep. 2023;13(1):8505. doi: 10.1038/s41598-023-35787-0 37231182 PMC10213040

[46] Ali A, Saeed S, Hussain R, Afzal G, Siddique AB, Parveen G, et al. Synthesis and Characterization of Silica, Silver-Silica, and Zinc Oxide-Silica Nanoparticles for Evaluation of Blood Biochemistry, Oxidative Stress, and Hepatotoxicity in Albino Rats. ACS Omega. 2023;8(23):20900–11. doi: 10.1021/acsomega.3c01674 37332821 PMC10269246

[47] Dube E, Okuthe GE. Engineered nanoparticles in aquatic systems: Toxicity and mechanism of toxicity in fish. Emerging Contaminants. 2023;9(2):100212. doi: 10.1016/j.emcon.2023.100212

[48] Sudhabose S, Sooryakanth B, Rajan MR. Acute Toxicity, Hematological Profile, and Histopathological Effects of MgO Nanoparticles on Gills, Muscle, Liver of Mrigal, Cirrhinus mrigala. Biol Trace Elem Res. 2024;202(2):736–42. doi: 10.1007/s12011-023-03704-1 37231319

[49] Mahmood NI Y, Maheen A, Mustafa G, Abdullah Bafail D, Qamar MR, Ahsan MA, et al. Multi-biomarker approach to assess oxidative stress and antioxidants profile in male albino rats exposed to ZnO nanoparticle. Asian Journal of Agriculture and Biology. 2024;2024(4).

[50] Afzal G, Ullah M, Ali N, Nizami M, Hussain R, Alhakamy N. Mechanistic approach to investigate the induction of toxicity by magnesium oxide nanoparticles on testicular, nervous and muscular tissues of albino rats. Asian Journal of Agriculture and Biology. 2024;50.

[51] Dingding L, Yan Y. Effects of Electroacupuncture on Behavioral deficits, Hippocampal Neuronal Death and Oxidative Stress in Rats with Parkinson’s Disease. Kafkas Universitesi Veteriner Fakultesi Dergisi. 2024;30(5).

[52] Olugbodi JO, Lawal B, Bako G, Onikanni AS, Abolenin SM, Mohammud SS, et al. Effect of sub-dermal exposure of silver nanoparticles on hepatic, renal and cardiac functions accompanying oxidative damage in male Wistar rats. Sci Rep. 2023;13(1):10539. doi: 10.1038/s41598-023-37178-x 37386048 PMC10310751

[53] Ullah A, Al-Saeed FA, Abduallah AM, Ahmed AE, Shahzad A, Amjad N, et al. Calcium Nanoparticles Induce Oxidative Stress in Erythrocytes, Neurotoxicity and Testicular Toxicity in Albino Rats (Rattus norvegicus). 2023.

[54] Alshammari GM, Al-Ayed MS, Abdelhalim MA, Al-Harbi LN, Yahya MA. Effects of Antioxidant Combinations on the Renal Toxicity Induced Rats by Gold Nanoparticles. Molecules. 2023;28(4):1879. doi: 10.3390/molecules28041879 36838869 PMC9959587

[55] Ghorbani S, Moshtaghi H, Shekarforoush S, Gheisari H, Sedaghati F, Nazifi S. Histopathologic, biochemical, and biodistribution studies of orally administrated silica and magnesium oxide nanoparticles in rats. Iranian Journal of Science. 2023:1–11.

[56] Kalsoom R, Asfour H, Ali H, Qayyum A, Anjum S, Maqbool F. Bifenthrin induced toxic effects on haematological, reproductive and histo-morphological profile in adult male quail (Coturnix japonica). Asian J Agric & Biol. 2024(4).

[57] Elshenawy OH, El-Kadi AOS. Modulation of aryl hydrocarbon receptor-regulated enzymes by trimethylarsine oxide in C57BL/6 mice: In vivo and in vitro studies. Toxicol Lett. 2015;238(1):17–31. doi: 10.1016/j.toxlet.2015.06.1646 26144063

[58] Zhang Y, Zhang E, Hou L, Lu H, Guo T, Wang R, et al. Assessing and mitigating foodborne acetochlor exposure induced ileum toxicity in broiler chicks: The role of omega-3 polyunsaturated fatty acids supplementation and molecular pathways analysis. Pestic Biochem Physiol. 2024;199:105761. doi: 10.1016/j.pestbp.2023.105761 38458672

[59] Memon AM, Kaka U, Umer M, Kamboh AA, Behan AA, Janyaro H, et al. Benefits of Incorporating Atipamezole in Medetomidine-ketamine Anaesthesia in Pigeons. PJZ. 2021;53(6). doi: 10.17582/journal.pjz/20200106110149

